# Measuring arousal and valence generated by the dynamic experience of architectural forms in virtual environments

**DOI:** 10.1038/s41598-022-17689-9

**Published:** 2022-08-04

**Authors:** Paolo Presti, Davide Ruzzon, Pietro Avanzini, Fausto Caruana, Giacomo Rizzolatti, Giovanni Vecchiato

**Affiliations:** 1 Institute of Neuroscience, National Research Council of Italy, 43125 Parma, Italy; 2 Department of Medicine and Surgery, University of Parma, 43125 Parma, Italy; 3 TUNED, Lombardini22, 20143 Milan, Italy; 4 Dipartimento Culture del Progetto, IUAV, 30125 Venice, Italy

**Keywords:** Human behaviour, Cognitive neuroscience

## Abstract

The built environment represents the stage surrounding our everyday life activities. To investigate how architectural design impacts individuals' affective states, we measured subjective judgments of perceived valence (pleasant and unpleasant) and arousal after the dynamic experience of a progressive change of macro visuospatial dimensions of virtual spaces. To this aim, we developed a parametric model that allowed us to create 54 virtual architectural designs characterized by a progressive change of sidewalls distance, ceiling and windows height, and color of the environment. Decreasing sidewalls distance, ceiling height variation, and increasing windows height significantly affected the participants' emotional state within virtual environments. Indeed, such architectural designs generated high arousing and unpleasant states according to subjective judgment. Overall, we observed that valence and arousal scores are affected by all the dynamic form factors which modulated the spaciousness of the surrounding. Showing that the dynamic experience of virtual environments enables the possibility of measuring the emotional impact of macro spatial architectural features, the present findings may lay the groundwork for future experiments investigating the effects that the architectural design has on individuals' mental state as a fundamental factor for the creation of future spaces.

## Introduction

A crucial but largely unexplored issue of human experience concerns how affective states are influenced by the dynamical change of spatial features when walking through a built environment. Previous studies using static 2D representations showed that several architectural features massively impact the observer's affective states, typically measured in valence and arousal^1^.

Valence represents the extent to which an architectural space makes an occupant feel good or bad. In recent studies, participants reported higher beautiful judgments for 2D representations of architectures with high ceilings and open spaces^2,3^, which were also perceived as more pleasant^4^. The presence of windows is also typically linked to a pleasant sensation^5^, enabling an outdoor view and creating a more spacious perception of the environment^6,7^. Moreover, cold colors typically receive higher valence ratings than warm ones, thus moving people's preferences towards cold environments^8–10^.

The arousal level can be modulated by the amount of light penetrating through the windows, which promotes circadian rhythms and contributes to people's overall well-being, diminishing their stress level^11^. Furthermore, cold-colored environments were associated with a peaceful sensation, while warm colors generated higher arousing states^10,12^. Previous research also reported that enclosed spaces increase vulnerability to stress and prolong the occupant's stress response^13^, while spaces with low ceilings were found to generate a sense of confinement, thus being associated with higher arousing states^14,15^.

However, most of the studies mentioned above referred to 2D representations of the architecture, i.e., pictures of architectures shown on a monitor screen, which barely generate a realistic architectural experience. Nowadays, virtual reality technologies are gaining importance in recreating immersive scenarios^16,17^, thus increasing the ecology of the architectural experience. Among such devices, head-mounted displays (HMDs) permit one to visually explore the surrounding space through a subjective camera that follows the head movement and consequently updates the sensory perception in real-time. This mechanism creates a place illusion^18^, generating realistic affective and behavioral responses in people, thus effectively providing a "reality simulator"^17^. Compared with 2D stimuli, immersive virtual reality simulates the environment in a realistic and possibly interactive modality^5^, ameliorates the spatial perception^19^, and can generate neurophysiological responses as a real scenario^20,21^. Nowadays, virtual reality is considered a powerful device for evoking emotions in laboratory settings^22–25^ compared to the presentation of passive stimuli^26^. Indeed, virtual reality potentiates emotion and attention processes compared to conventional 2D stimulation, ensuring a deeper emotional experience^27–29^. Virtual reality has also been exploited in architecture to investigate the occupant emotions exposed to different architectural features^22,30,31^. Also, immersive virtual environments generated similar behavioral outcomes compared to physically built environments^32–34^. Hence, by adopting this technology, it is possible to effectively investigate the emotional perception of architecture under controlled laboratory conditions^5,30,35^.

In particular, previous studies showed that walking within immersive 3D spaces significantly modulated individuals' emotional states. Ziegelman and colleagues found that different virtual scenarios elicited changes in state anxiety during walking^36^. Walking within virtual parks with different emotional contents elicited the intended emotions and a high sense of presence^37^. Also, immersive scenarios induced relaxing states while participants could freely navigate around^38,39^.

Although virtual reality has gained significant interest in investigating the architectural experience in the last years, only a few studies exploited its potential to permit a dynamic perception of the surrounding space^40,41^. However, these works test the perception of affordances through the design of several architectural transitions (i.e., walking through a door of a different dimension), comparing static versus dynamic conditions, not revealing emotional aspects, or investigating the emotional impact of static architectural forms not generating a dynamic perception of the surrounding^42^.

To overcome these limitations, we exploited the virtual reality technology to make subjects dynamically experience a set of immersive environments, conceived as a progressive modulation of the surrounding space only characterized by macro spatial dimensional changes, thus without any extraneous element.

Considering that exploring the space is fundamental for a comprehensive perception of the environment^43^, we exploited the HMD to enable an immersive, dynamic architecture experience. The subjective camera moves within the virtual architectural design to dynamically perceive the surrounding space, thus generating a walking sensation. Indeed, previous research found that moving the user's view from one point to another can provide a compelling sense of self-motion^44–46^. Such strategy allowed subjects to look around and see the space from different perspectives and positions, which is fundamental for visually exploring and perceiving the environment in a natural way^42^. Also, we minimized the presence of contextual elements within the virtual architectural designs. Indeed, they were conceived as a progressive modulation of the surrounding space, with no extraneous elements, thus only characterized by macro spatial dimensional changes. Such dimensional changes were varied in a controlled way, thus creating 54 different environments where specific architectural parameters were manipulated either separately or in a combined way. Specifically, we manipulated the progressive variation of architectural elements such as sidewall distances, ceiling height, sill height, and color. Subjects made a virtual promenade within the environment and then rated their experienced valence and arousal level.

This study demonstrates that a progressive modulation of spaciousness impacts the dynamic experience of architectural forms, thus influencing the subjective affective states^47,48^. In line with permeability theories^49^, our results show that moving towards narrow spaces with a reduced possibility to look around and visually explore the surrounding generates anguishing sensations. Showing that the dynamic perception of macro spatial architectural changes influences individuals' affective states, this study fosters a design approach that posits human affective states at the core of the creation of future spaces. We conclude with several possible future perspectives and implications, showing how the proposed experimental framework could be exploited to acquire a more generalizable knowledge of the architectural experience.

## Material and methods

### Participants

Twenty-nine subjects were recruited for this experiment (27.35 ± 3.7 years, 16 female). All subjects were Italian master's or postgraduate students. They had no particular familiarity with virtual reality, and none of them was an architect, studied architecture, or was in any way familiar with the study of architecture, internal design, and related disciplines. The sample size was determined using a power analysis computed through the G*Power software^50^, considering the 2 × 3 × 3 × 3 within-subject design with the "as in SPSS" option and setting the significance level (α) at 0.05, the desired power (1 − β) to 0.95, the number of groups to 1, the number of repetition to 54 and the non-sphericity correction ɛ to 1. The value of the η_p_^2^ was set to 0.03 based on previous research^4^. All subjects reported normal or corrected-to-normal vision and no previous history of neurological disorders. The study was approved by the local ethical committee (Comitato Etico dell’Area Vasta Emilia Nord, https://www.aou.mo.it/ComitatoEticoAVEN), and conducted according to the principles expressed in the Declaration of Helsinki. Each participant provided written informed consent before participating in the experiment.

### Stimuli

A parametric model was designed on Grasshopper (https://www.rhino3d.com/6/new/grasshopper/) and imported into the online platform ShapeDiver (https://shapediver.com/) to generate 27 different virtual architectural designs. Each environment is a combination of three consecutive nuclei, the first of which had fixed dimensions: 4 m × 4 m × 7 m (width × height × length) with 1 m × 6.8 m (height × length) windows located in the middle height of each sidewall. Then, the other two nuclei could vary architectural factors such as the distance between the sidewalls (SideWalls), the height of the ceiling (Ceiling), and the height of the windows sill (Windows), which refers to the location of the windows relative to the ceiling height. Specifically, the parametric model allowed increasing, decreasing, and keeping such architectural factors constant between one nucleus and the next. Thus, we realized variations of ± 0.8 m for SideWalls and Ceiling factors. The sill height changed as a function of the ceiling height of the third nucleus: in the increasing sill height condition, windows were located close to the ceiling; in the decreasing condition, windows were located close to the floor; in the constant condition, windows were located in the middle of the sidewalls. The sill height of the second nucleus was defined considering the middle point between the sill height of the first and third nucleus. All virtual designs were endowed with an open door at the entrance and a closed one at the end. Such configuration enhanced the realism of the dynamic experience of the architecture, giving the subject the impression of being inside an environment suitable for a promenade, providing an entry and an exit point. The dimensions of the architectural designs are shown in the Supplementary Material.

**Figure 1:**
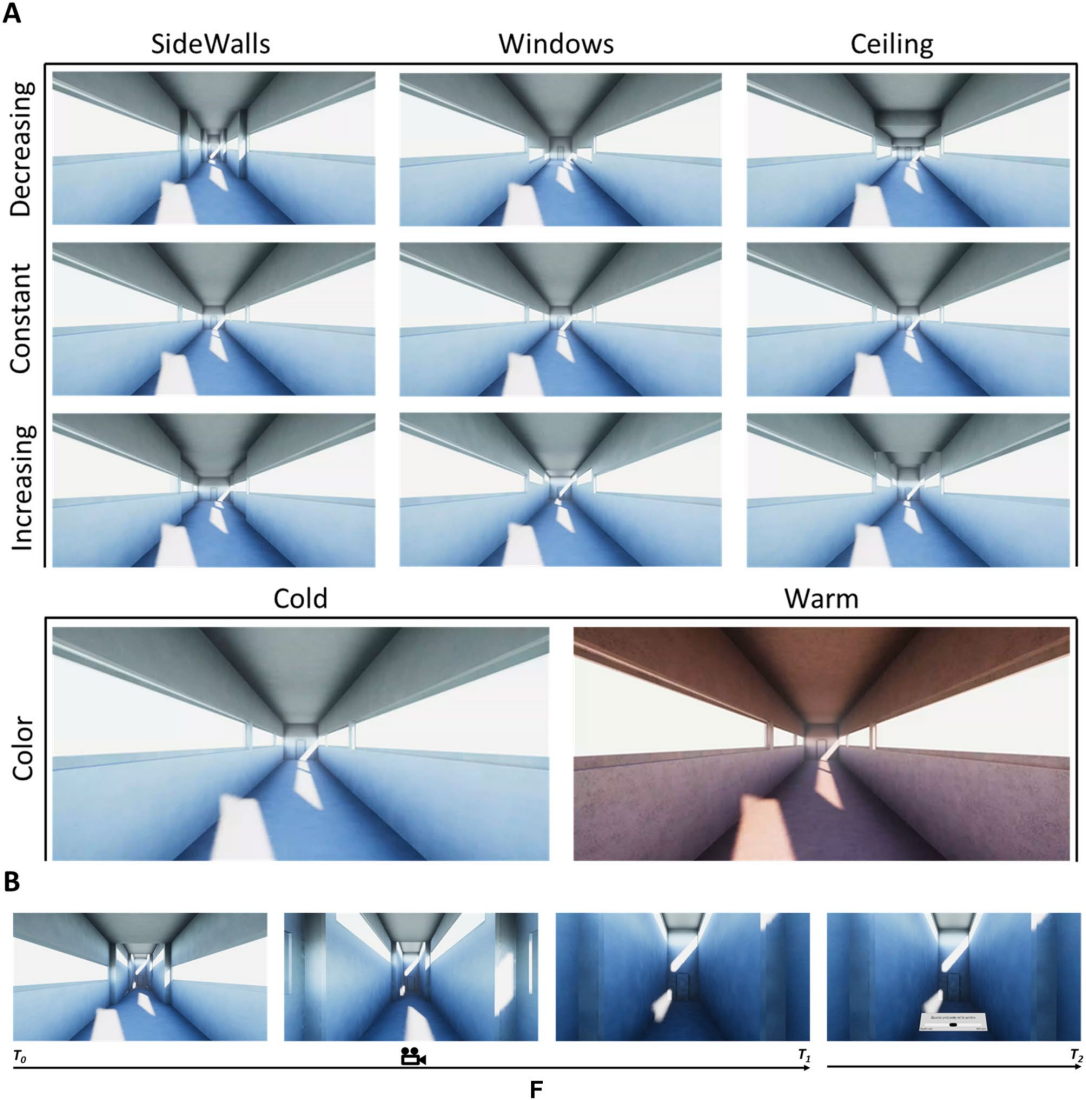
(**A**) Perspectives of virtual environments for each architectural factor (SideWalls, Ceiling, Windows, Color) for the corresponding experimental conditions (decreasing, constant, increasing; cold, warm). (**B**) schematic representation of one experimental trial: subject made a virtual promenade between the first two nuclei of the architectural design (T0-T1) and then rated the experienced level of arousal and pleasantness (T2).

The 3D architectural designs generated in Shapediver were imported into Unity (2019.1.0f2, https://unity.com/). Here, parameters such as texture and lighting were manipulated to transform the 3D object into a realistic architecture. Hence, each design was proposed in two colors: a warm reddish and cold bluish. A specific lighting setting was implemented to generate realistic enlightenment of the virtual scene. The light was a directional one 4 m high, located 2.5 m to the right and 4.5 m before the entrance door outside the environment. The light was also inclined towards the door, 18° around the x-axis (width) and − 17° around the y-axis (height), making the lighting assume different shapes depending on architectural factors. In this way, 54 architectural designs were created to implement a 3 × 3 × 3 × 2 factorial model with factors SideWalls × Ceilings × Windows (each with conditions decreasing, constant, increasing) x Colors (warm, cold). Figure 1A provides a view of the architectural factors from the entrance perspective.

### Experimental setup

The experiment was performed in a virtual reality environment realized with the HTC Vive Pro Eye HMD to provide a highly immersive experience. It is equipped with two AMOLED screens, with a resolution of 1440 × 1600 pixels per eye, a refresh rate of 90 Hz, and a field of view of 110°. Unity was integrated into the HMD via the Steam VR asset to control the experimental procedure and collect data. The experiment ran on a laptop with Windows 10 Home (64-bit), Intel Core i7 -9750H, 32 GB RAM, and the NVIDIA GeForce RTX 2070 graphics card.

### Experimental procedure

Subjects read written instructions explaining the experimental procedure and the concepts of valence and arousal. Valence is the pleasantness state generated by a given experience: unpleasant states are associated with bad feelings or a negative state of mind, while pleasant states with good feelings or a positive state of mind^51–56^. The arousal dimension is the state of activation generated by a given experience, resembling a change in the individual's physical and psychological assets. A deactivated state is associated with a low heartbeat, sweating decrease, slow breathing, absence of energy, and decreased attentional and decisional capability. Instead, an activated state is associated with a high heartbeat, sweating increase, fast breathing, feelings of vigor, energy, tension, and increasing attentional and decisional capability^51,56,57^. The Vive Pro Eye HMD was placed over the subject's head and arranged comfortably, providing a clear view of the virtual environment. Through the movement of a first-person perspective camera, subjects experienced the feeling of walking within the architecture. Specifically, the navigation within the environment was realized by moving the camera's position from the entrance door to the beginning of the third nucleus. Hence, the subjective camera was moved within the virtual environment, following the subject's head rotation and displacement (6 degrees of freedom), thus ensuring the elicitation of the place illusion. The camera speed was set to 1.25 m/s, considering the average male and female gait speed^58^. Also, a smoothing function was applied to avoid rough speed changes at the beginning and the end of the navigation, thus limiting and preventing motion sickness.

Thus, each experimental trial consisted of a virtual promenade (12.5 s) inside the environment, followed by the presentation of two consecutive grey panels with an approximation of a visual analog scale (VAS) where subjects could rate the architectural experience in terms of arousal and valence. The lower and higher bound of the two VAS were 0 and 1, respectively. With the first scale, subjects answered the question: "This environment makes me feel…", ranging from "Deactivated" to "Activated". With the second scale, subjects answered the question: "This environment provides me … feelings", ranging from "Unpleasant" to "Pleasant". Subjects used the Vive controller to move the VAS cursor without time limits to provide their judgments. The 54 architectural designs were randomly presented, divided into three blocks of 18 environments each, thus subjects experienced each design once. Blocks were separated by a pause in which subjects were permitted to take the HMD off and have some rest. At the beginning of the experiment and during each pause, the experimenter ensured that subjects did not feel any motion sickness effects. No subjects reported motion sickness during the experiment. Figure 1B depicts the timeline of an experimental trial.

### Statistical analysis

Subjective valence and arousal ratings were z-scored and analyzed via two distinct 3 × 3 × 3 × 2 repeated measures ANOVA (rmANOVA) with SideWalls, Ceiling, Windows (decreasing, constant, increasing), and Colors (warm, cold) as within-subject factors. Pearson's correlation coefficient was used to compute the correlation between arousal and valence scores. K-means clustering (k = 2) was used to group the environments in the space defined by the arousal and valence dimensions. For each of the four environmental factors, we computed the prevalence of the architectural design for each experimental condition. Finally, the statistical difference in the observed frequency distribution was assessed by chi-squared tests. Data analysis was performed with Matlab 2018b (The Mathworks, Inc., Natick, MA, USA) and Statistica 7 (StatSoft Europe) software.

## Results

**Figure 2:**
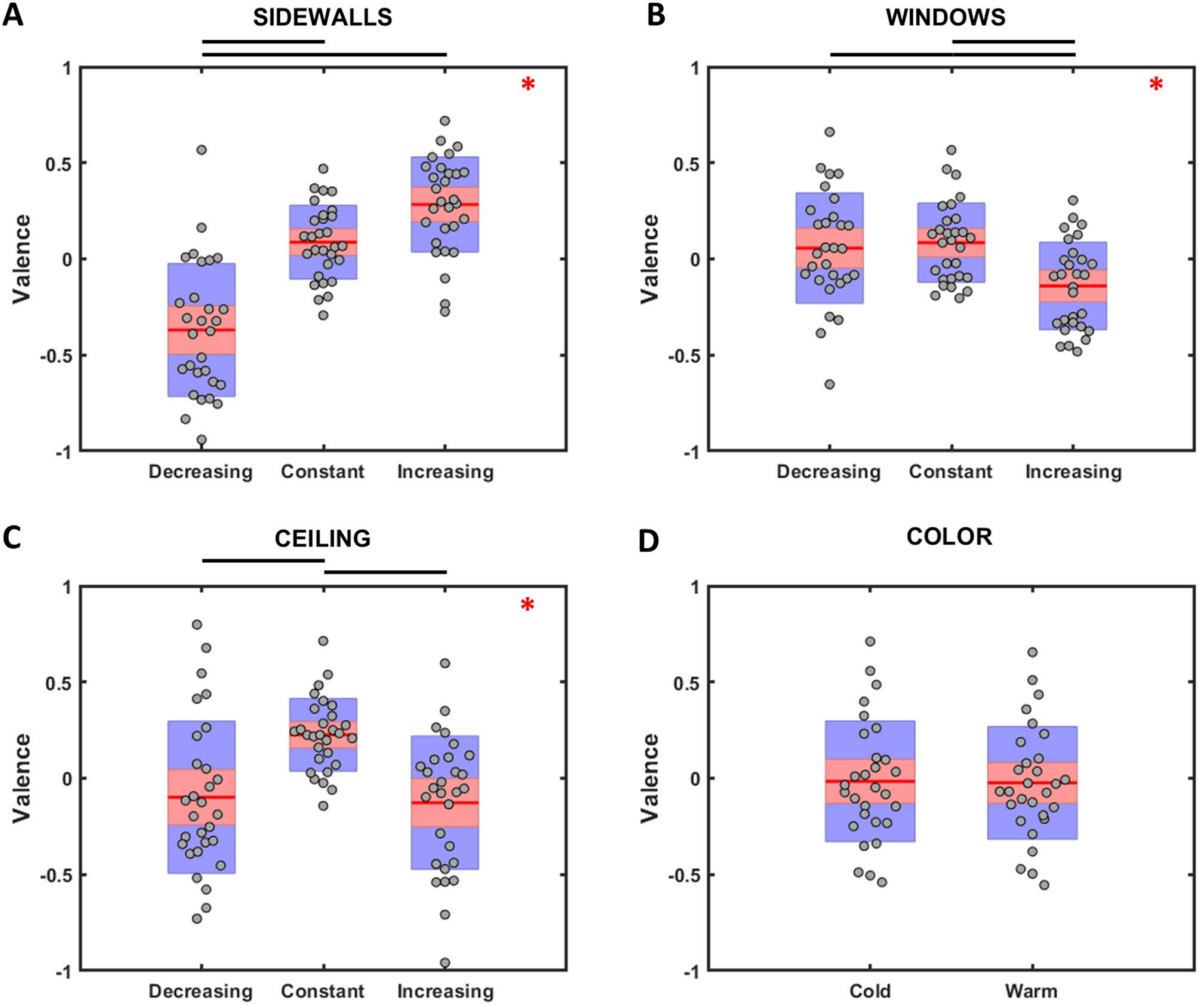
Distribution of the valence scores for SideWalls, Windows, Ceiling, and Color are presented in (**A**–**D**), respectively. Red lines represent the mean value; data are represented as dots laid over a 1.96 standard error mean (95% confidence interval) in pink and a 1 standard deviation in blue. Red asterisks indicate a significant main effect, and black lines show significant pairwise comparisons.

**Figure 3:**
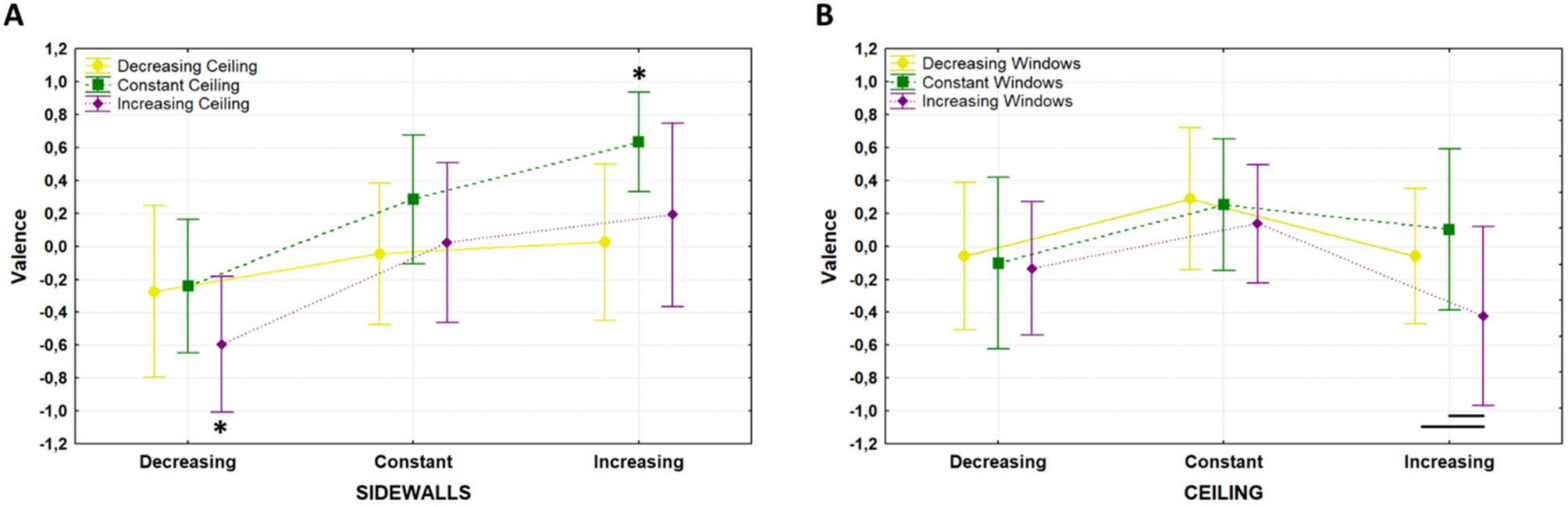
(**A**) distribution of the valence scores for the two-way interaction SideWalls × Ceiling. Yellow, green, and purple lines represent decreasing, constant, and increasing ceiling height. (**B**) distribution of the valence scores for the two-way interaction Ceiling × Windows. Yellow, green, and purple lines represent decreasing, constant, and increasing windows height. Data are presented with their mean value with vertical lines representing the 95% confidence interval. Black lines stand for significant pairwise comparison, while black asterisks remark an experimental condition that all comparisons with the other conditions are significant.

Results of the rmANOVA on valence ratings are illustrated in Fig. 2. A significant effect was observed for the main factors SideWalls (F(2,56) = 29.933, p = 1.4 * 10^–9^, η_p_^2^ = 0.516), Windows (F(2,56) = 4.941, p = 0.011, η_p_^2^ = 0.149) and Ceiling (F(2,56) = 7.135, p = 0.002, η_p_^2^ = 0.203). Specifically, Bonferroni corrected pairwise comparisons revealed lower valence ratings for those architectural designs with decreasing sidewalls distance when compared to those with constant (p = 6.6 * 10^–6^) and increasing (p = 1.3 * 10^–9^) one. Increasing windows sill height also resulted in lower valence scores compared to the constant (p = 0.016) and decreasing (p = 0.044) conditions. Finally, designs with constant ceiling height were associated with higher valence ratings than those with increasing (p = 0.003) and decreasing one (p = 0.008). No significant difference among valence scores was observed for the factor Color. The interaction SideWalls × Ceiling (F(4,112) = 6.219, p = 1.5 * 10^–4^, η_p_^2^ = 0.182) was significant as illustrated in Fig. 3A. Bonferroni corrected pairwise comparisons revealed that architectural designs with increasing sidewalls distance and constant ceiling height resulted in significantly higher valence scores than designs with different combinations concerning the sidewalls distance and ceiling height. Contrarily, those architectural designs with decreasing sidewalls distance and increasing ceiling height received significantly lower valence scores than those with different combinations of such architectural elements. Also the interaction Windows x Ceiling (F(4,112) = 3.297, p = 0.013, η_p_^2^ = 0.105) returned to be significant as presented in Fig. 3B. Specifically, Bonferroni corrected pairwise comparisons revealed that subjects provided significant lower valence scores for those designs with increasing windows height rather than decreasing (p = 0.022) or constant (p = 5 * 10^–5^), but only when the ceiling of the environment increased between one nucleus and the next one, and not in the decreasing or constant ceiling height condition.

**Figure 4:**
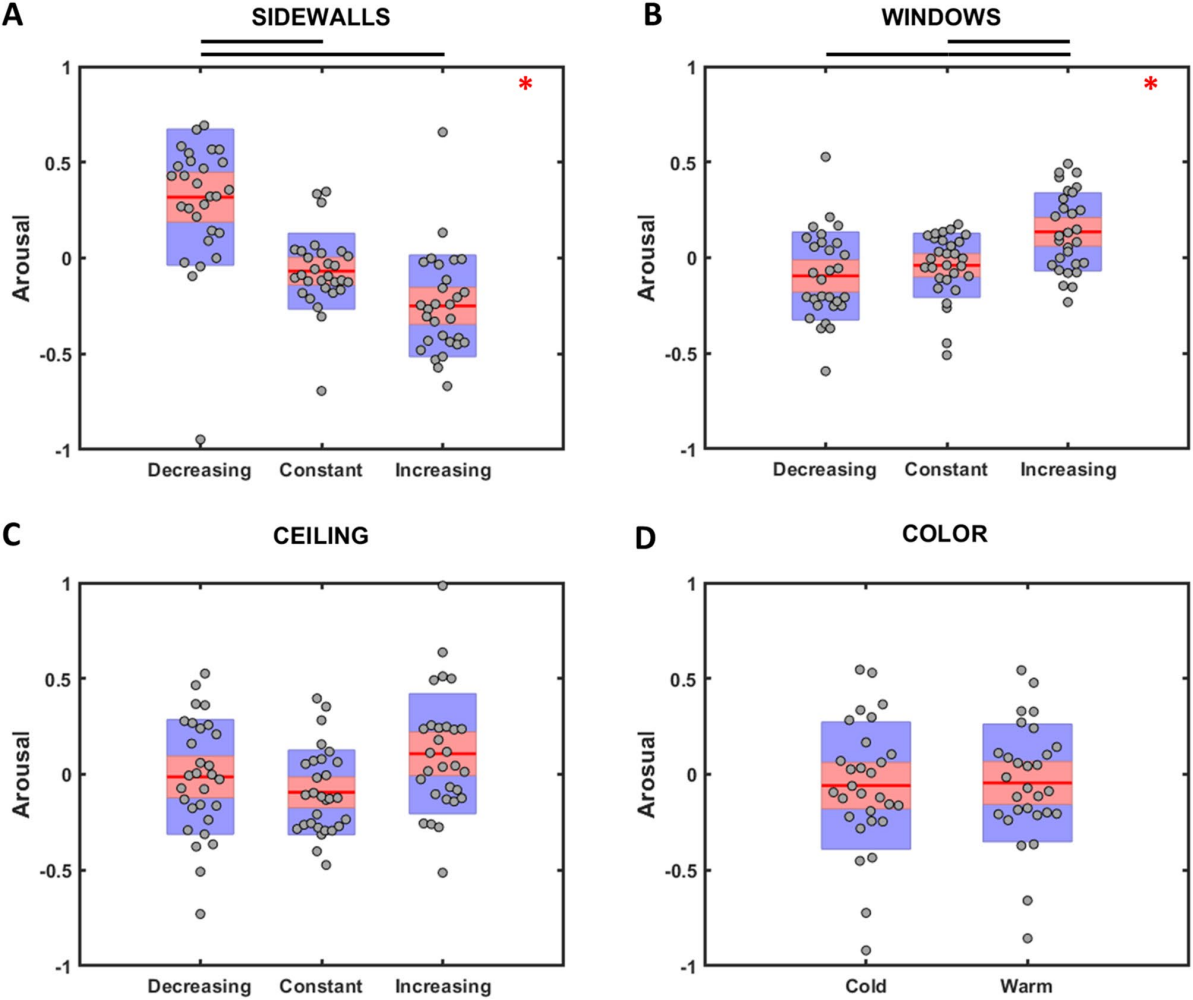
Distribution of arousal scores for the main factors SideWalls, Windows, Ceiling, and Color are presented in (**A**–**D**), respectively. Same color code as Fig. 2.

**Figure 5:**
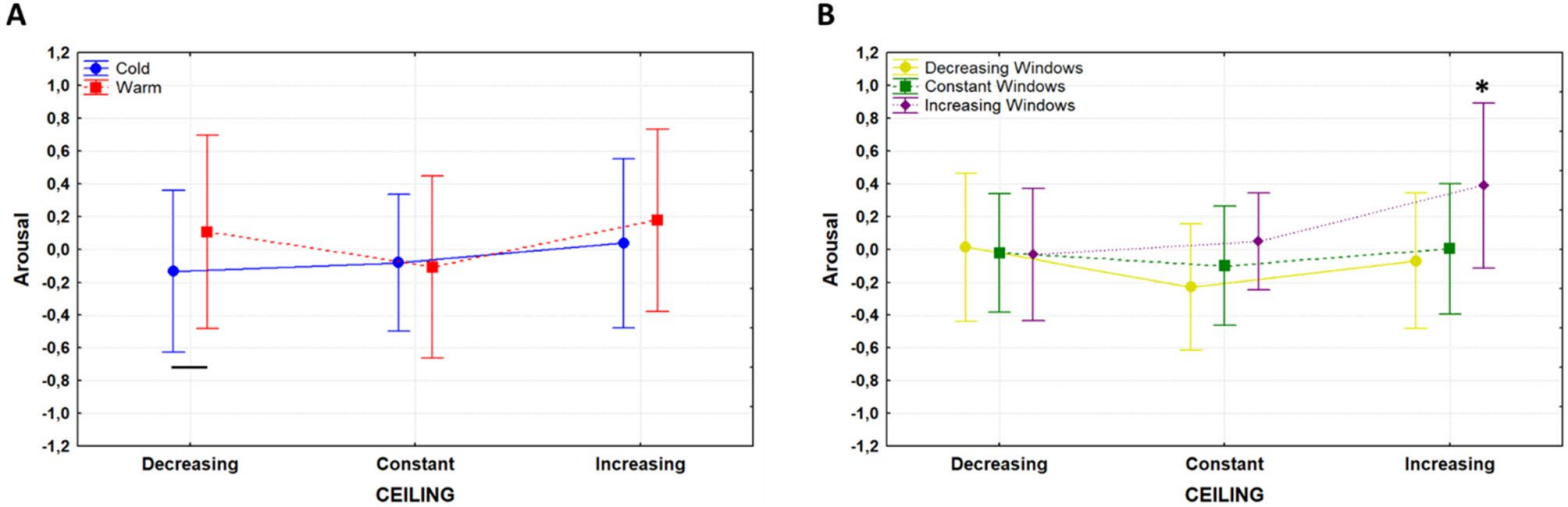
(**A**) distribution of the arousal scores for the two-way interaction Color × Ceiling. Blue and red lines represent cold and warm architectural designs, respectively. (**B**) Distribution of the arousal scores for the two-way interaction Windows x Ceiling, same color code as Fig. 3.

Results of the rm ANOVA on arousal ratings are illustrated in Fig. 4. A significant effect was found for the main factors SideWalls (F(2,56) = 20.589, p = 1.9 * 10^–7^, η_p_^2^ = 0.424) and Windows (F(2,56) = 6.812, p = 0.002, η_p_^2^ = 0.196), with a trend for the factor Ceiling (F(2,56) = 2.519, p = 0.089). Specifically, Bonferroni corrected pairwise comparisons revealed that architectural designs with decreasing sidewalls distance resulted in higher arousal scores than those with constant (p = 2.2 * 10^–4^) and increasing (p = 1.5 * 10^–7^) sidewalls distance. Designs with increasing windows sill height were perceived as more arousing than those with constant (p = 0.029) and decreasing ones (p = 0.002). No significant difference among the arousal scores was observed for the factor Color. A significant interaction of the factor Color × Ceiling (F(2,56) = 4.23, p = 0.019, η_p_^2^ = 0.131) is illustrated in Fig. 5A. Bonferroni corrected pairwise comparison revealed that subjects perceived warm environments as more arousing than cold ones when the ceiling decreased between consecutive nuclei (p = 0.008). The interaction Windows × Ceiling (F(4,112) = 3.747, p = 0.007, η_p_^2^ = 0.118) was also significant (Fig. 5B), showing that higher arousal scores were collected in architectural designs with increasing windows height and increasing ceiling, compared to those designs with different combination of such architectural features.

**Figure 6:**
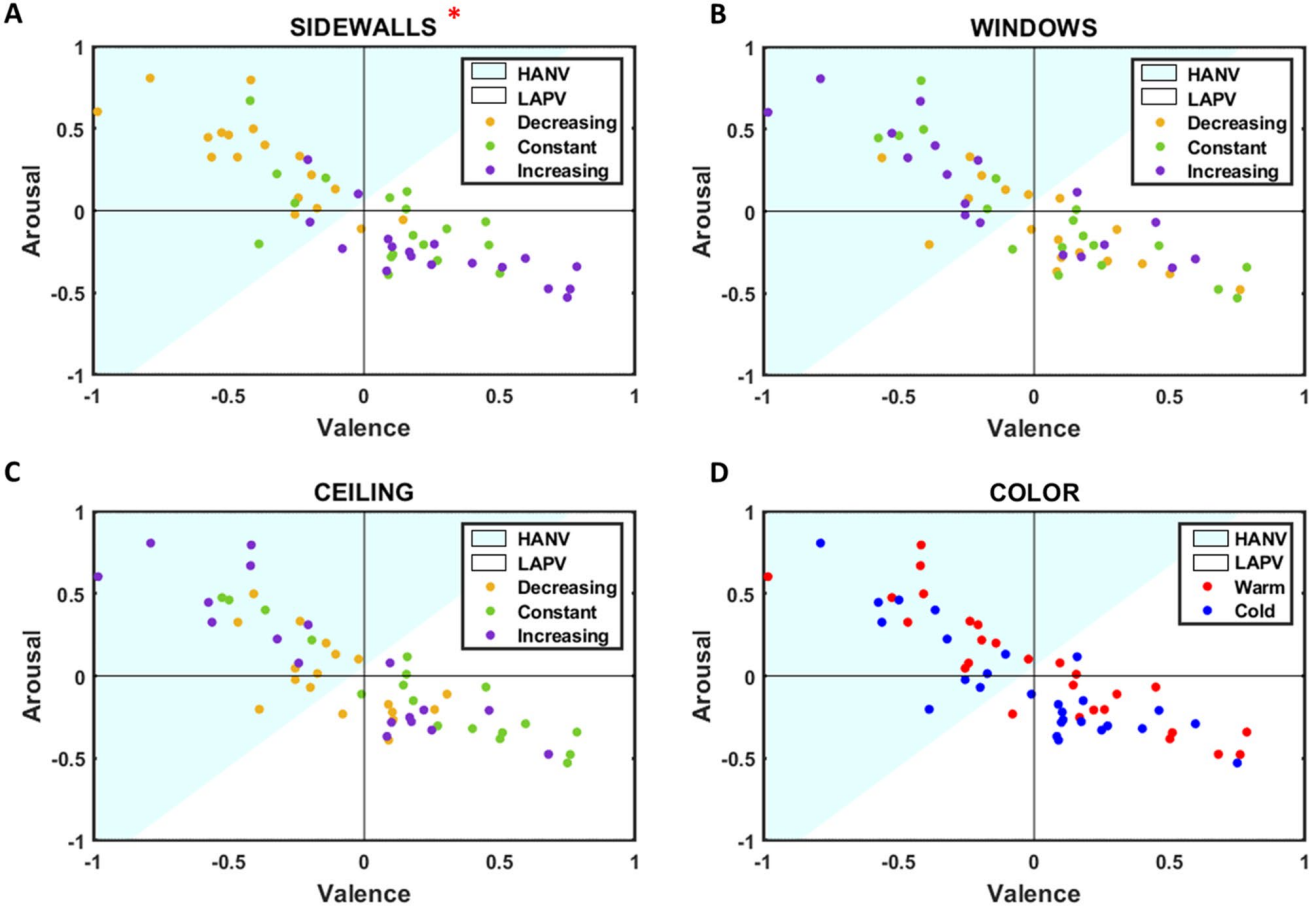
For each panel, the blue section of the plane includes the architectural designs belonging to the HANV cluster, while the white one comprises those designs within the LAPV cluster. (**A**–**C**) Yellow, green, and purple dots identify environments with decreasing, constant and increasing conditions for SideWalls, Windows, and Ceiling factors, respectively. (**D**) Blue and red dots identify architectural designs with cold and warm texture colors, respectively. The red asterisk indicates statistically significant results.

The results of the cluster analysis are illustrated in Fig. 6. Architectural designs are distributed in the space defined by arousal and valence dimensions with a significant negative correlation (R = − 0.85, p < 0.001). Hence, we applied the k-means algorithm (k = 2) to segregate the environments experienced with a high level of arousal and a negative level of valence (HANV) from those experienced with low arousal and positive valence (LAPV). The prevalence of designs within the two clusters according to their experimental condition can be found in Table 1. Considering the factor SideWalls, we observed that environments were largely unbalanced between the two clusters (χ^2^ = 20.05, p = 1.6 * 10^–5^): the 89% of designs with decreasing sidewalls distance belong to the HANV cluster, while most of the constant and increasing sidewalls distance designs belong to the LAPV cluster (72 and 83%). Furthermore, we found that the distribution of the environments within the two clusters according to the ceiling height was close to being significant (χ^2^ = 5.85, p = 0.054): the 78% of designs with constant ceilings belong to the LAPV cluster, while the 61% of designs with decreasing ceiling belong to the HANV cluster. According to the factors Windows and Color, clustering architectural designs did not return statistically significant results.

**Table 1:** Percentage of architectural design distribution within the clusters HANV and LAPV for each experimental condition.

		HANV	LAPV
SIDEWALLS	Decreasing	88.89	11.11
	Constant	27.78	72.22
	Increasing	16.67	83.33

		HANV	LAPV
Windows	Decreasing	38.89	61.11
	Constant	33.33	66.67
	Increasing	61.11	38.89

		HANV	LAPV
Ceiling	Decreasing	61.11	38.89
	Constant	22.22	77.78
	Increasing	50	50

		HANV	LAPV
Color	Warm	48.15	51.85
	Cold	40.74	59.26

## Discussion

We used virtual reality technology to create a virtual promenade, which was experienced emotionally through macro spatial dimensional changes of the environments formed by three consecutive nuclei whose architectural features varied progressively. The proposed experimental framework allowed us to isolate the effects of macro spatial architectural changes on subjects' affective states, finding that negative-valenced feelings were generated by narrowing the sidewalls, increasing the windows sill height, or increasing/decreasing the ceiling height. In the first two cases, such modulations also significantly increased perceived arousal. Rm ANOVAs and cluster analysis revealed that the architectural feature that more strongly affected valence and arousal ratings was the sidewalls distance. Finally, we found that our architectural spaces generated either pleasant and low arousing states or unpleasant and high arousing states, possibly arguing that macro spatial architectural changes may be associated with relaxing or anguishing spaces and rarely with pleasant and exciting spaces or unpleasant and calming ones.

Our results showed that subjects preferred to experience the virtual promenade within wide spaces rather than enclosed ones, arguing that the progressive reduction of the surrounding space was perceived as a constriction, thus leading to uncomfortable states of unpleasantness and high arousal^47,48^. These results align with enclosure and permeability theories, according to which enclosed spaces, characterized by a reduced possibility to move-through and see-through, are associated with uncomfortable states^49^. Previous studies also revealed that 2D stimuli of enclosed spaces were more likely to generate fear sensation and avoidance decisions and judged as less beautiful than open spaces^2,3,59^. Furthermore, enclosed spaces are associated with situations perceived as less controllable and avoided by human beings because they increase the stress level of their inhabitants^13^. Reasons can be found in survival motivation since enclosed spaces do not typically provide any possible way out^49,60^.

The virtual promenade characterized by decreasing ceiling height produced a progressive reduction of the surrounding space, thus leading subjects to report unpleasant judgments. We found a preference peak for the constant ceiling height of 4 m, resembling results favoring built environments with a 3 m ceiling height that fosters exploration and visuospatial attention^3,4,61^. Such a 1 m difference concerning the reported literature could be due to general misperception effects generated by virtual reality^62,63^. Findings suggest that egocentric distances in virtual environments are estimated as 75% of the modeled virtual distance^64^. Instead, the over-increasing ceiling height led to perceiving the architectural space as less pleasant, as also reported by Baird and colleagues^61^, possibly due to a decreased perception of the spaciousness^15^.

We report that virtual promenades with increasing windows sill height generated unpleasant and arousing judgments. In such architectural spaces, light penetrated from progressively higher and less accessible points, leading subjects to move towards less enlightened areas where a possible outdoor view was more challenging to access. Previous research claimed that these aspects are fundamental for the well-being of the inhabitants^65^. Although we cannot measure the impact of the virtual simulation of sunlight on circadian rhythms, the realistic enlightenment we created within the virtual architectural designs contributed to creating the "place illusion," thus leading to the consequent modulation of affective states. In addition, access to outdoor views was found to reduce the stress level at the work office^66^, provide restoration at home^67^, and benefit post-operative patients during recovery^68^. The presence of windows is also typically associated with an increased perception of the spaciousness of the environment^7,69^. In line with such findings, our results emphasize that moving towards spaces where the windows are closer to the subject's height produced a more pleasant experience with lower arousal values, possibly due to an increased perception of the spaciousness^11,70,71^. The proposed experimental design showed that participants felt an anguishing sensation within those architectural designs with increasing sill height relative to the ceiling, regardless of the absolute height of the sill. As shown in the Supplementary Material, we verified if the eyes’ position could contribute to these individual preferences with no significant results. Hence, further investigation is needed to study how the relative height between the eye level and the sill contributes to modifying the individuals' affective states with proper experimental conditions.

The environment's color modulation did not affect valence and arousal judgments at the end of the dynamic architectural experience. The specific bluish and reddish colors were selected to generate cold and warm sensations. To more deeply investigate how the chromatic aspect of the environment modulates the individual emotional state, we need to consider additional chromatic characteristics, such as the different values of hue, intensity, and saturation, possibly perceived in different modalities. For instance, one could introduce a dynamic component to the chromatic characteristics that could change across the nuclei, as done with the form factors, to investigate the role of a dynamic component of the color on emotional judgments. On the other hand, one could represent the environments statically to compensate for the dynamic component generated by changes in the macro spatial architectural dimensions, thus hypothesizing that a static color could mainly influence a static, not dynamic, perception of the environment.

These results suggest that modification of forms produces a variation of the extra-personal space, which the subject can visually explore, thus affecting the perception of spaciousness. Since it is known that space coding relies on both visual as well as motor circuits^72–75^, we may hypothesize that the emotional experience generated by the dynamic perception of progressive variation of architectural features could exploit the neural circuitry composed of parietal and premotor areas devoted to controlling and planning of voluntary movements^76–79^.

This study demonstrates the capability of virtual reality technologies to evaluate individuals' emotional response to key spatial architectural parameters experienced during a virtual promenade. On the one hand, virtual reality itself does have some caveats related to the limited sensorial stimulation and thus to the transferability of the results into real-world architectures. A comprehensive architectural experience is characterized by a larger variety of sensorial aspects, rather than only visual, that may interact to ultimately shape individuals' affective states. On the other hand, virtual reality has the unique advantage of allowing the creation of ecologically valid experimental scenarios without confounding effects present in real-world investigations.

One challenge faced by virtual reality technology is reproducing a realistic perception of illuminance and colors. In earlier studies, actual reference rooms were compared to their full-scale reconstructed virtual models. Virtual rooms presented incorrect light reflections on surfaces and several issues related to chromatic and color characteristics (i.e., achromatic shadows, limited contrast effects, and limited color variations)^80,81^. In the last years, important steps forward have been made regarding color rendering in 3D virtual models, permitting to recreate a more realistic illuminance, pruned by color-related issues^82^. Indeed, recent studies compared individuals' responses to real architectures and their virtual reproduction, finding no significant differences. In a study by Chamilothori and colleagues, they found that using immersive virtual environments is an adequate surrogate of real-world settings in daylight perceptual studies^83^. Latini and colleagues compared subjects' productivity and comfort between real and immersive virtual scenarios of an office considering different color layouts finding no statistical differences^32^.

The objective of the present study was to demonstrate that the only dynamic perception of macro visuospatial variations of forms could alter the affective experience of the environment, regardless of other sensorial variables. For this reason, we exploited virtual reality to render a series of environments with different visuospatial effects while keeping constant all the other architectural components, such as material, that would have impacted—without the possibility to test—the other sensorial channels. In addition, it could also be worth considering that vision is dominant over the other senses. In this regard, it was demonstrated that sensorial experiences of touch are negligible when coupled with visual stimulation to evaluate different materials and their warmth perception^84,85^.

The generalizability of the present findings should be tested across different cultural backgrounds. Indeed, perception of spaciousness and color preferences may vary according to the cultures^86–88^. Generally, westerners are more focused on salient stimulus features independently of their surrounding spatial or social context^89,90^. Saulton and colleagues showed that German and South Koreans had different spatial volume perceptions of computer-generated rooms. Specifically, they found that Koreans were significantly less biased than Germans by room rectangularity and viewpoint, pointing to the necessity of further investigations to determine the exact reasons for the cultural differences in room size perception^88^. Also, color preferences may vary according to different cultures. For instance, Eastern Europeans were significantly more prone to green colors, while Western Europeans to dark greys^91^. Similarly, Jonauskaite and colleagues found that the yellow-joy association was more frequent in participants who lived far away from the equator and in rainier countries^92^. As a future perspective, our experimental framework offers the possibility to test cultural differences by ad-hoc manipulating forms and colors of the environment according to theories and findings reported in the literature, thus providing a valid approach to investigate why people from different ethnicities and cultures perceive architecture differently.

The adopted framework will also allow recreating immersive scenarios where the architecture is the context hosting our everyday activities and where individuals interact in social scenarios. Indeed, humanoid avatars can be embedded within virtual settings, thus reproducing those events that typically occur within built environments. In that case, one will be able to investigate how the architecture shapes social interactions, which are otherwise challenging to recreate in a standard laboratory setting or natural conditions.

In addition, behavioral and physiological measures could be coupled with the resulting explicit subjective reports to investigate the neural underpinnings and the corresponding actions emerging from the dynamic perception of architecture^35,42,93^. Eye-tracking systems could be embedded into the HMD to gather eye gaze and pupillometry for a description related to the patterns of the visuospatial exploration. The recording of autonomic parameters could be synchronized with the virtual stimulation system to couple physiological measures related to emotional processes. Finally, electroencephalography can be recorded during the virtual reality experience to monitor cortical circuits underpinning the dynamic perception of architecture. In such a way, physiological data would reveal the implicit correlates of the emotional experience elicited by the perception of a progressive modulation of the extra-personal space associated with the generation of relaxing and anguishing states.

## Conclusions

The present study demonstrates that the dynamic experience of architectural design macro spatial variations influences the individuals' affective state. The visuospatial exploration of those environments characterized by a progressive reduction of the extra-personal space generated anguishing sensations. Extending the proposed experimental framework to investigate physiological reactions to architectural changes will advance a more generalizable knowledge of architectural perception. Thus, the present findings may pave the way for future studies revealing the effects of the architectural experience on the behavioral and psychophysiological level of the human being in social contexts.

## Supplementary Information

Supplementary Information.

## Data Availability

The datasets collected and analyzed during the current study are available from the corresponding author on request.
