# Supplementary material for: Measuring arousal and valence generated by the dynamic experience of architectural forms in virtual environments
Source: Sci Rep. 2022 Aug 4;12:13376. doi: 10.1038/s41598-022-17689-9 (PMC9352685; doi:10.1038/s41598-022-17689-9)
Supplement: Supplementary file 1 — Supplementary Information. [file 41598_2022_17689_MOESM1_ESM.pdf]

Supplementary Material

Measuring arousal and valence generated by the dynamic experience of architectural forms in virtual environments

Paolo Presti[1,2], Davide Ruzzon[3,4], Pietro Avanzini[1], Fausto Caruana[1], Giacomo Rizzolatti[1] and Giovanni Vecchiato[1,*]

[1]Institute of Neuroscience, National Research Council of Italy, Parma, 43125, Italy
[2]Department of Medicine and Surgery, University of Parma, Parma, 43125, Italy
[3]TUNED, Lombardini22, Milan, 20143, Italy
[4]Dipartimento Culture del Progetto, IUAV, Venice, 30125, Italy
[*]giovanni.vecchiato@in.cnr.it

Material and methods

Stimuli

Supplementary Figure S1 illustrates the macro spatial dimensions that we manipulated to create the 27 different architectural designs.

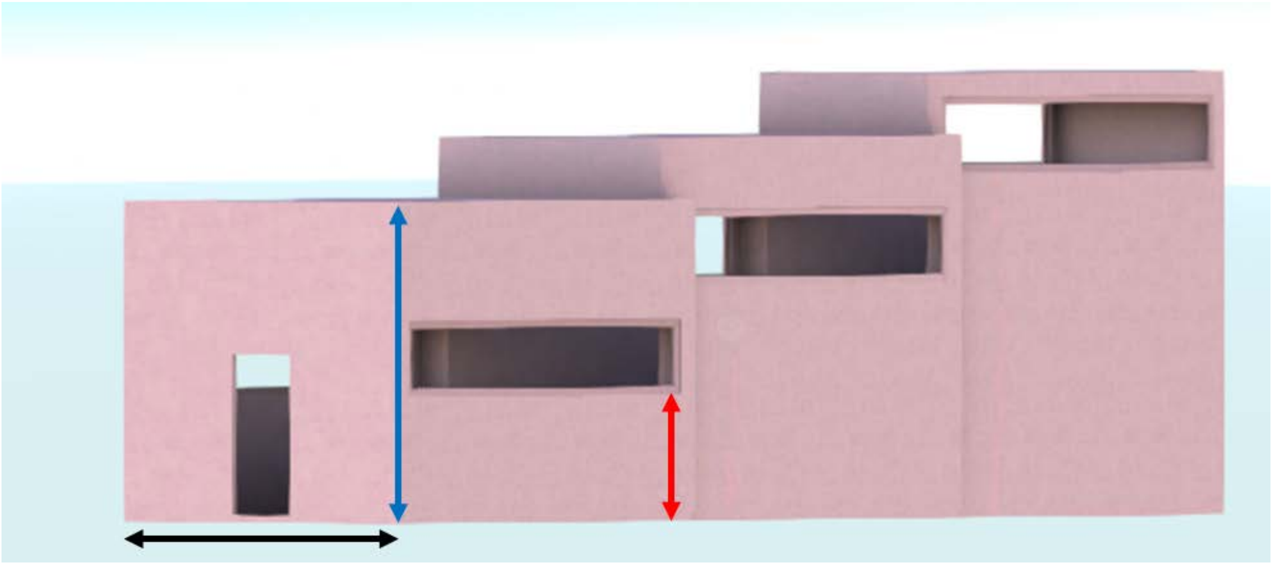

Figure S1. The architectural design with increasing sidewalls distance, increasing ceiling height and increasing sill height is presented here as example. The black arrow indicates the sidewalls distance, the blue one the ceiling height and the red one the sill height.

Each of the architectural design is illustrated in supplementary Figure S2 where values of sidewalls distance, ceiling height and sill height are reported for each nucleus within a table. Furthermore, a lateral view and a view from the top are shown for each architectural designs.

Nuceli Dimensions	Lateral View	Top View

	1° Nucleus	2° Nucleus	3° Nucleus
Side Wall Distance Increasing	4 m	4.8 m	5.6 m
Ceiling Height Increasing	4 m	4.8 m	5.6 m
Sill Height Increasing	1.5 m	2.95 m	4.4 m

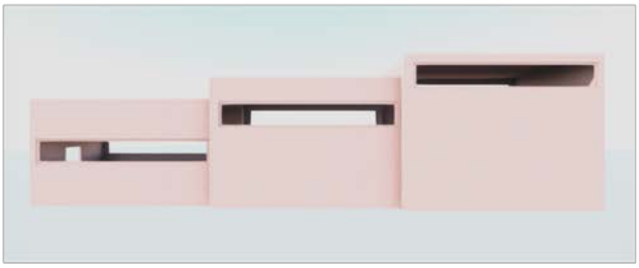

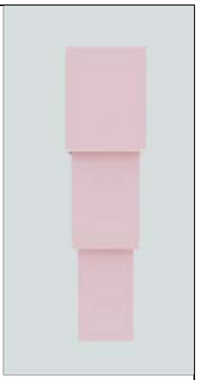

	1° Nucleus	2° Nucleus	3° Nucleus
Side Wall Distance Increasing	4 m	4.8 m	5.6 m
Ceiling Height Decreasing	4 m	3.2 m	2.4 m
Sill Height Increasing	1.5 m	1.35 m	1.2 m

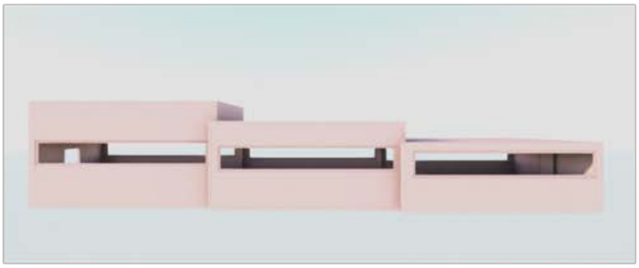

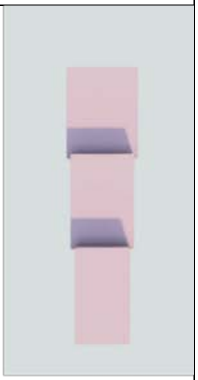

	1° Nucleus	2° Nucleus	3° Nucleus
Side Wall Distance Increasing	4 m	4.8 m	5.6 m
Ceiling Height Constant	4 m	4 m	4 m
Sill Height Increasing	1.5 m	2.15 m	2.8 m

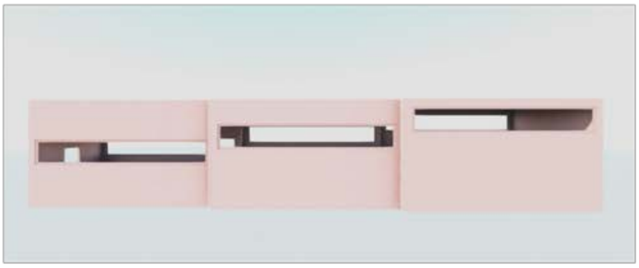

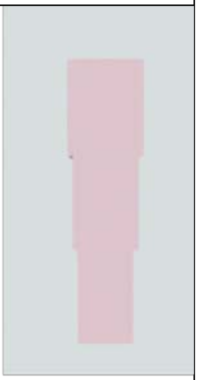

	1° Nucleus	2° Nucleus	3° Nucleus
Side Wall Distance Increasing	4 m	4.8 m	5.6 m
Ceiling Height Increasing	4 m	4.8 m	5.6 m
Sill Height Decreasing	1.5 m	0.85 m	0.2 m

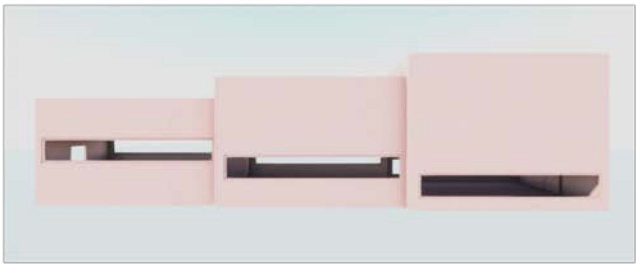

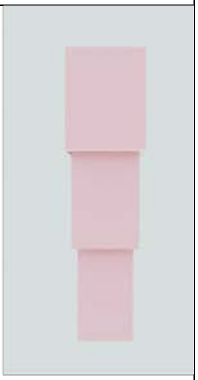

	1° Nucleus	2° Nucleus	3° Nucleus
Side Wall Distance Increasing	4 m	4.8 m	5.6 m
Ceiling Height Decreasing	4 m	3.2 m	2.4 m
Sill Height Decreasing	1.5 m	0.85 m	0.2 m

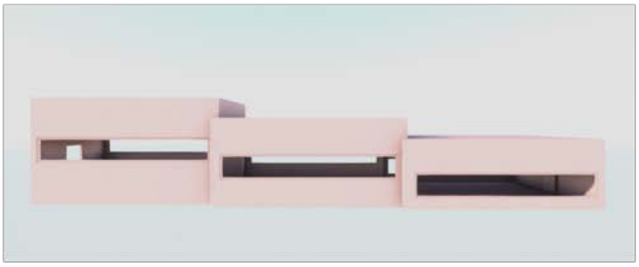

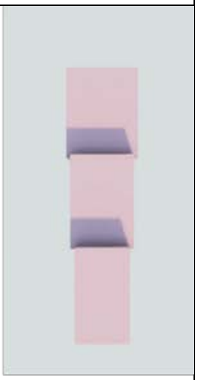

	1° Nucleus	2° Nucleus	3° Nucleus
Side Wall Distance Increasing	4 m	4.8 m	5.6 m
Ceiling Height Constant	4 m	4 m	4 m
Sill Height Decreasing	1.5 m	0.85 m	0.2 m

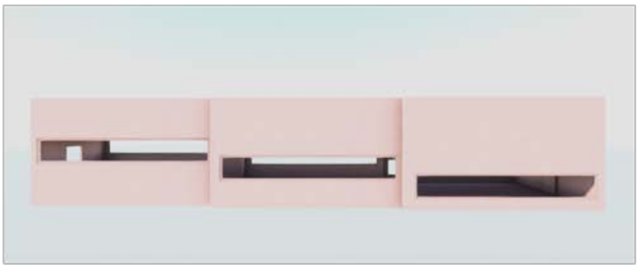

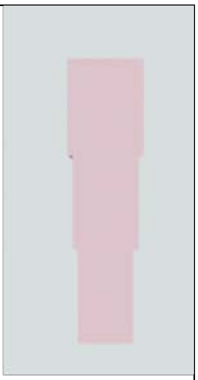

	1° Nucleus	2° Nucleus	3° Nucleus
Side Wall Distance Increasing	4 m	4.8 m	5.6 m
Ceiling Height Increasing	4 m	4.8 m	5.6 m
Sill Height Constant	1.5 m	1.9 m	2.3 m

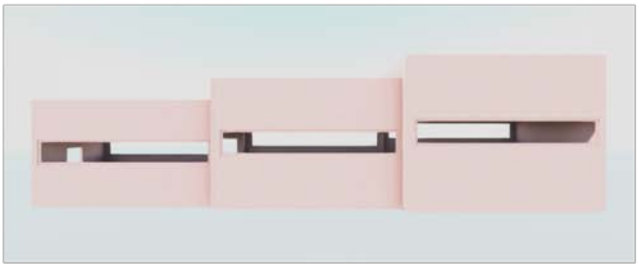

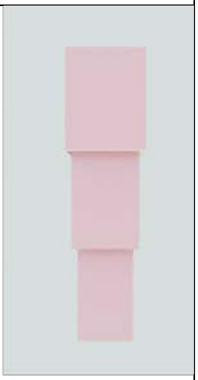

	1° Nucleus	2° Nucleus	3° Nucleus
Side Wall Distance Increasing	4 m	4.8 m	5.6 m
Ceiling Height Decreasing	4 m	3.2 m	2.4 m
Sill Height Constant	1.5 m	1.1 m	0.7 m

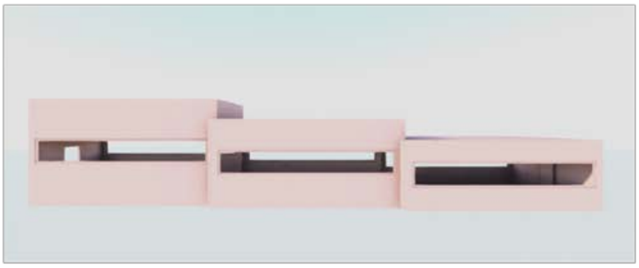

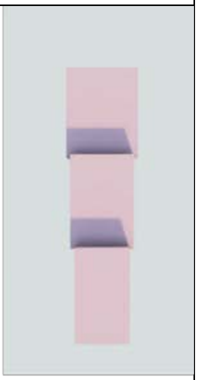

	1° Nucleus	2° Nucleus	3° Nucleus
Side Wall Distance Increasing	4 m	4.8 m	5.6 m
Ceiling Height Constant	4 m	4 m	4 m
Sill Height Constant	1.5 m	1.5 m	1.5 m

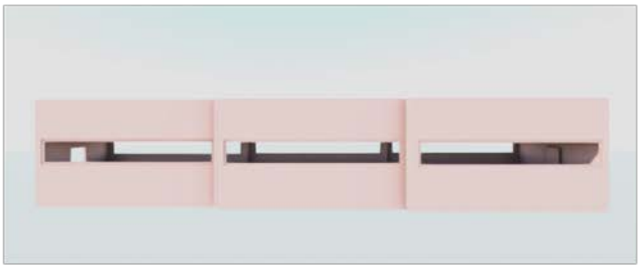

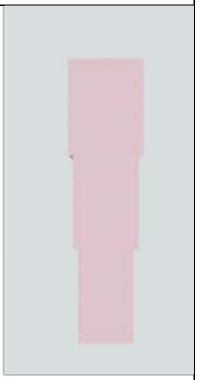

	1° Nucleus	2° Nucleus	3° Nucleus
Side Wall Distance Decreasing	4 m	3.2 m	2.4 m
Ceiling Height Increasing	4 m	4.8 m	5.6 m
Sill Height Increasing	1.5 m	2.95 m	4.4 m

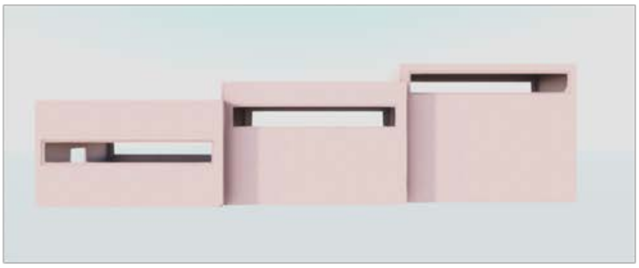

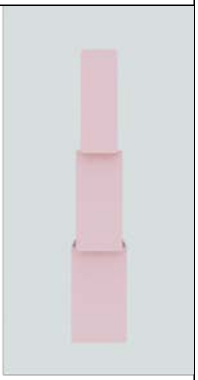

	1° Nucleus	2° Nucleus	3° Nucleus
Side Wall Distance Decreasing	4 m	3.2 m	2.4 m
Ceiling Height Decreasing	4 m	3.2 m	2.4 m
Sill Height Increasing	1.5 m	1.35 m	1.2 m

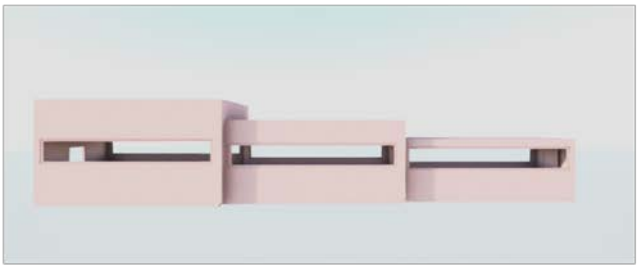

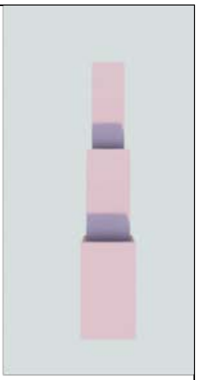

	1° Nucleus	2° Nucleus	3° Nucleus
Side Wall Distance Decreasing	4 m	3.2 m	2.4 m
Ceiling Height Constant	4 m	4 m	4 m
Sill Height Increasing	1.5 m	2.15 m	2.8 m

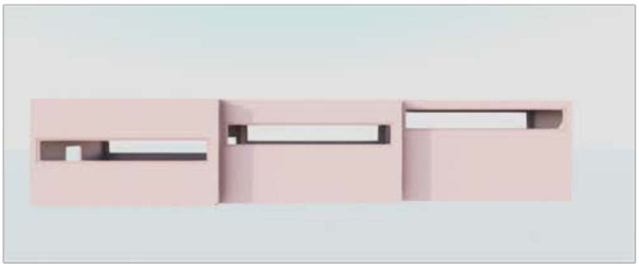

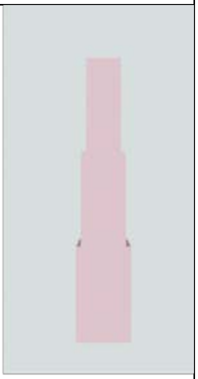

	1° Nucleus	2° Nucleus	3° Nucleus
Side Wall Distance Decreasing	4 m	3.2 m	2.4 m
Ceiling Height Increasing	4 m	4.8 m	5.6 m
Sill Height Decreasing	1.5 m	0.85 m	0.2 m

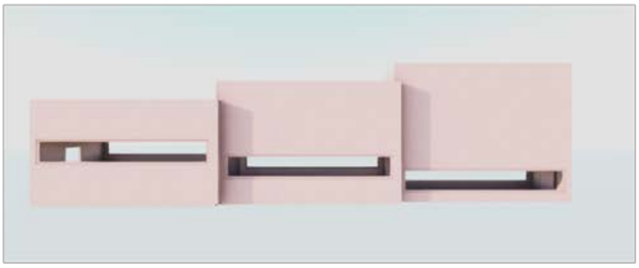

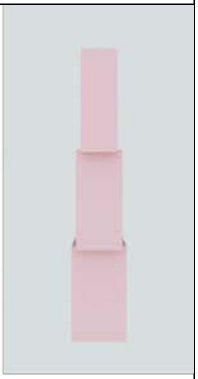

	1° Nucleus	2° Nucleus	3° Nucleus
Side Wall Distance Decreasing	4 m	3.2 m	2.4 m
Ceiling Height Decreasing	4 m	3.2 m	2.4 m
Sill Height Decreasing	1.5 m	0.85 m	0.2 m

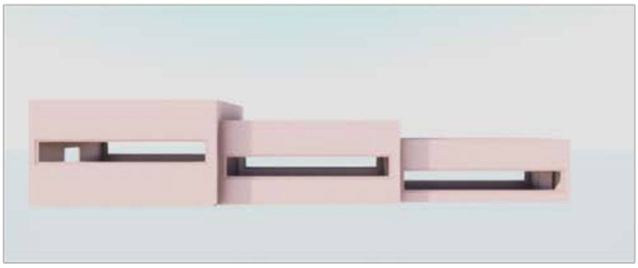

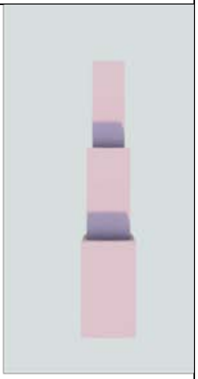

	1° Nucleus	2° Nucleus	3° Nucleus
Side Wall Distance Decreasing	4 m	3.2 m	2.4 m
Ceiling Height Constant	4 m	4 m	4 m
Sill Height Decreasing	1.5 m	0.85 m	0.2 m

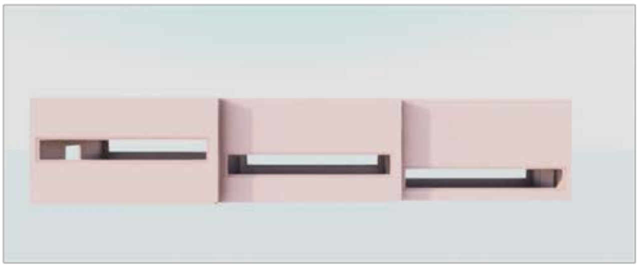

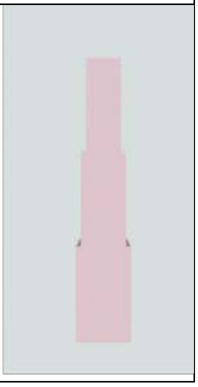

	1° Nucleus	2° Nucleus	3° Nucleus
Side Wall Distance Decreasing	4 m	3.2 m	2.4 m
Ceiling Height Increasing	4 m	4.8 m	5.6 m
Sill Height Constant	1.5 m	0.9 m	2.3 m

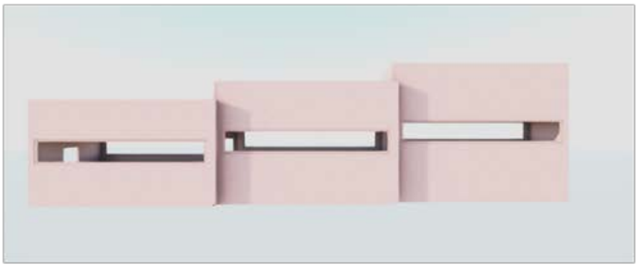

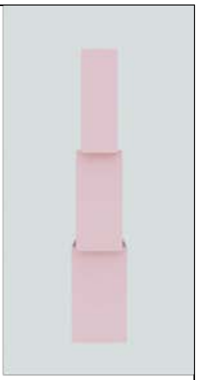

	1° Nucleus	2° Nucleus	3° Nucleus
Side Wall Distance Decreasing	4 m	3.2 m	2.4 m
Ceiling Height Decreasing	4 m	3.2 m	2.4 m
Sill Height Constant	1.5 m	1.1 m	0.7 m

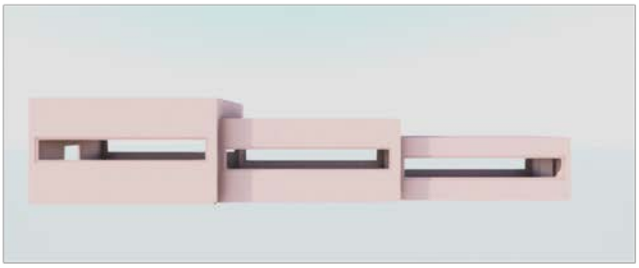

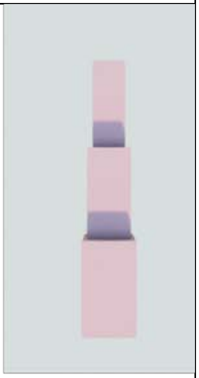

	1° Nucleus	2° Nucleus	3° Nucleus
Side Wall Distance Decreasing	4 m	3.2 m	2.4 m
Ceiling Height Constant	4 m	4 m	4 m
Sill Height Constant	1.5 m	1.5 m	1.5 m

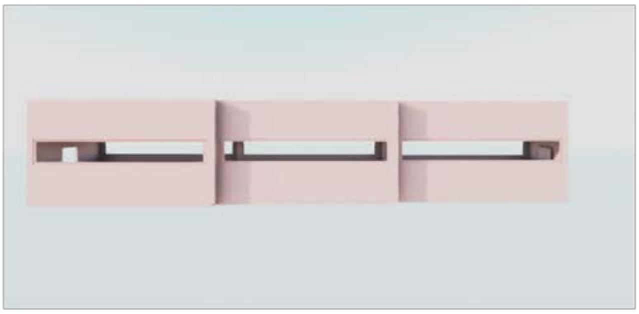

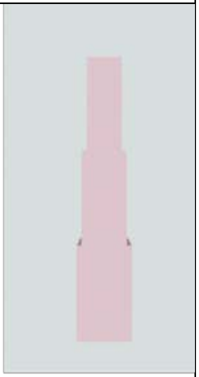

	1° Nucleus	2° Nucleus	3° Nucleus
Side Wall Distance Constant	4 m	4 m	4 m
Ceiling Height Increasing	4 m	4.8 m	5.6 m
Sill Height Constant	1.5 m	2.95 m	4.4 m

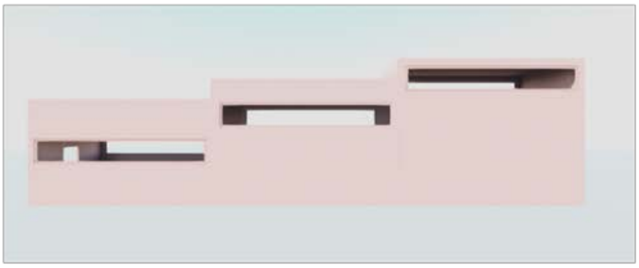

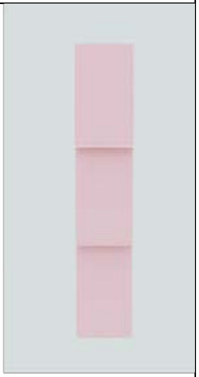

	1° Nucleus	2° Nucleus	3° Nucleus
Side Wall Distance Constant	4 m	4 m	4 m
Ceiling Height Decreasing	4 m	3.2 m	2.4 m
Sill Height Increasing	1.5 m	1.35 m	1.2 m

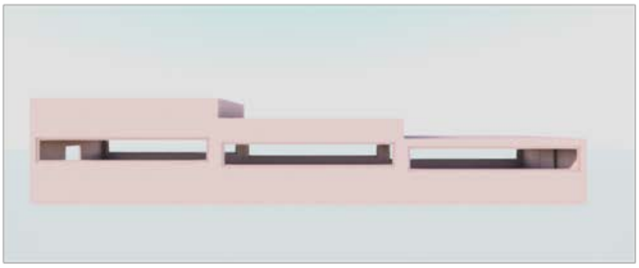

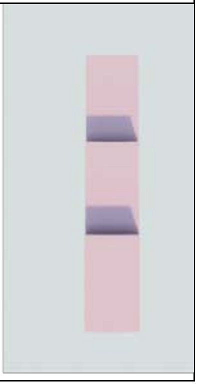

	1° Nucleus	2° Nucleus	3° Nucleus
Side Wall Distance Constant	4 m	4 m	4 m
Ceiling Height Constant	4 m	4 m	4 m
Sill Height Increasing	1.5 m	2.15 m	2.8 m

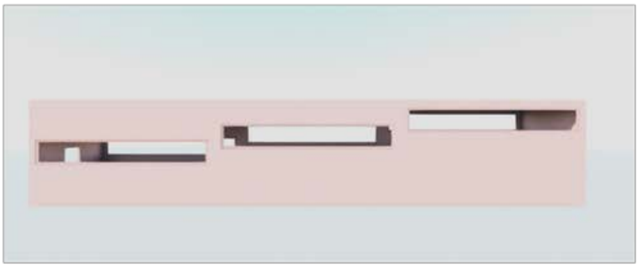

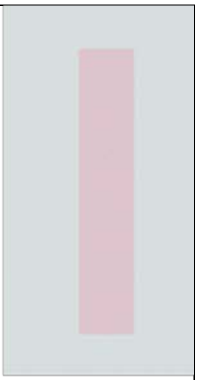

	1° Nucleus	2° Nucleus	3° Nucleus
Side Wall Distance Constant	4 m	4 m	4 m
Ceiling Height Increasing	4 m	4.8 m	5.6 m
Sill Height Decreasing	1.5 m	0.85 m	0.2 m

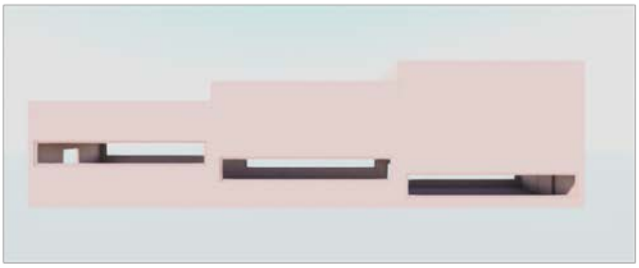

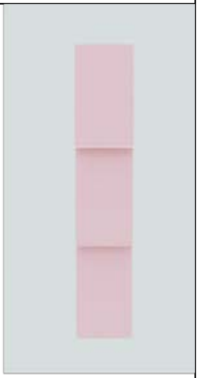

	1° Nucleus	2° Nucleus	3° Nucleus
Side Wall Distance Constant	4 m	4 m	4 m
Ceiling Height Decreasing	4 m	3.2 m	2.4 m
Sill Height Decreasing	1.5 m	0.85 m	0.2 m

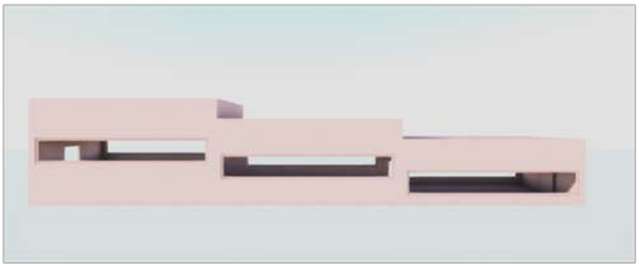

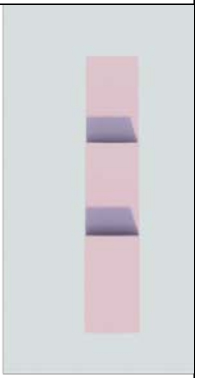

	1° Nucleus	2° Nucleus	3° Nucleus
Side Wall Distance Constant	4 m	4 m	4 m
Ceiling Height Constant	4 m	4 m	4 m
Sill Height Decreasing	1.5 m	0.85 m	0.2 m

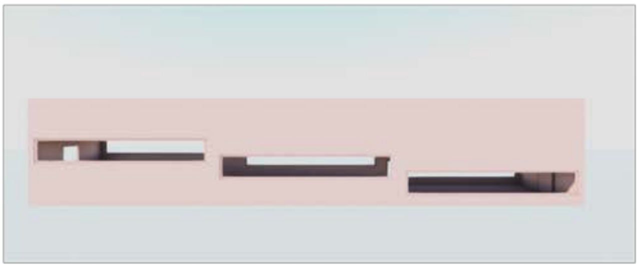

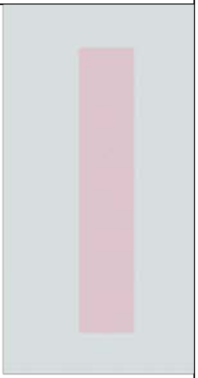

	1° Nucleus	2° Nucleus	3° Nucleus
Side Wall Distance Constant	4 m	4 m	4 m
Ceiling Height Increasing	4 m	4.8 m	5.6 m
Sill Height Constant	1.5 m	1.9 m	2.3 m

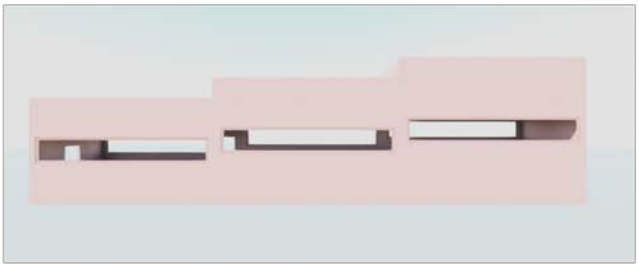

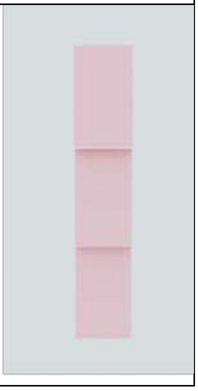

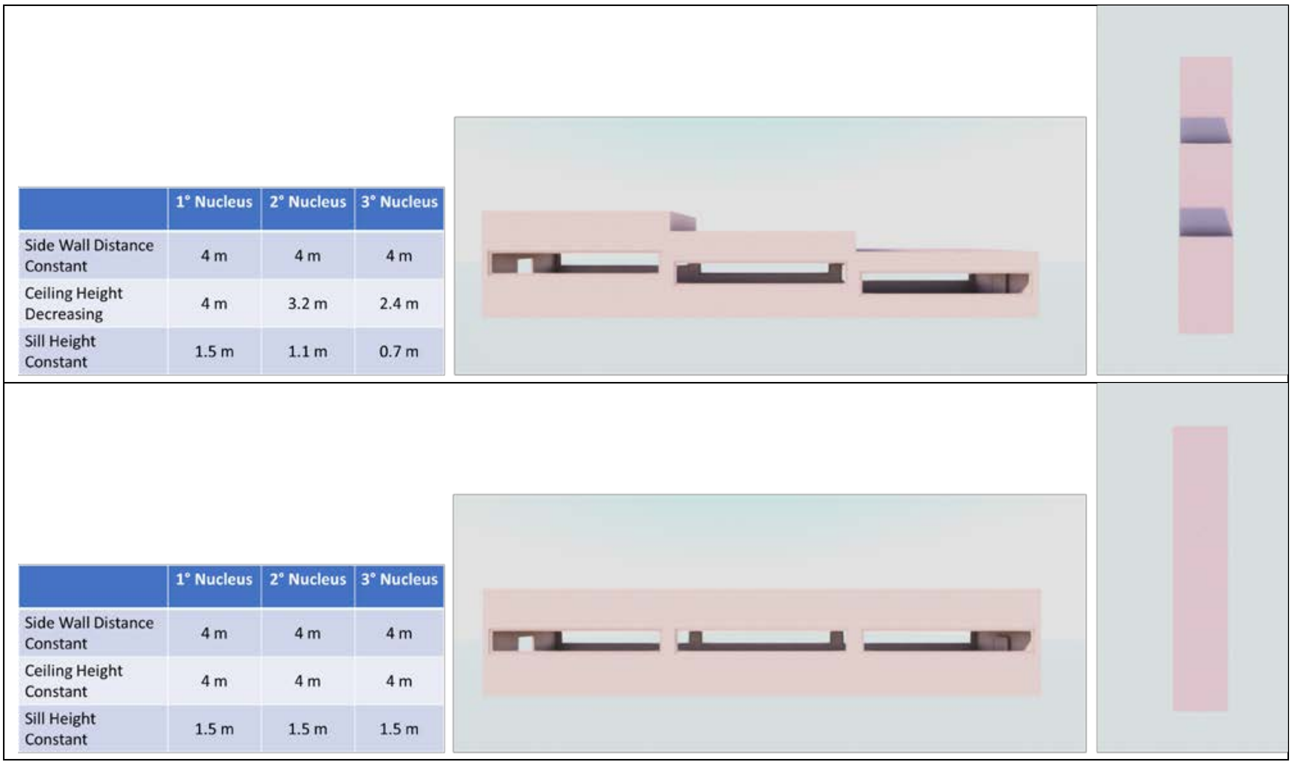

	1° Nucleus	2° Nucleus	3° Nucleus
Side Wall Distance Constant	4 m	4 m	4 m
Ceiling Height Decreasing	4 m	3.2 m	2.4 m
Sill Height Constant	1.5 m	1.1 m	0.7 m

	1° Nucleus	2° Nucleus	3° Nucleus
Side Wall Distance Constant	4 m	4 m	4 m
Ceiling Height Constant	4 m	4 m	4 m
Sill Height Constant	1.5 m	1.5 m	1.5 m

Figure S2. The tested architectural designs are illustrated in the figure. Within each row we represent a different design. Specifically, from left to right, we show: i) a table with the dimensions of the sidewalls distance, ceiling height and sill height for each nucleus of the design; ii) a lateral view of the design; iii) a top view of the design.

Results

A preliminary correlation analysis was computed between the average brightness measured within the virtual architectures and subjective z-scored valence and arousal ratings. Indeed, due to differences in terms of colors and forms, virtual architectures were characterized by different brightness values. To compute the brightness of each architecture, we firstly recorded a digital video of the virtual promenade inside the architecture. Then, the brightness was computed as the mean value of the RGB triplets relative to each video frame. Hence, we performed two separate Pearson's correlation analyses to test the independence between the brightness and subjective scores of arousal and valence.

Figure S3 showed the results of the correlation analysis between the brightness of the architectures and valence ($R = -0.09$, $p = 0.52$) and arousal ($R = 0.07$, $p = 0.62$) subjective ratings. No correlation was found, highlighting that the affective modulation generated by the architectural experience did not depend on such a low-level brightness factor.

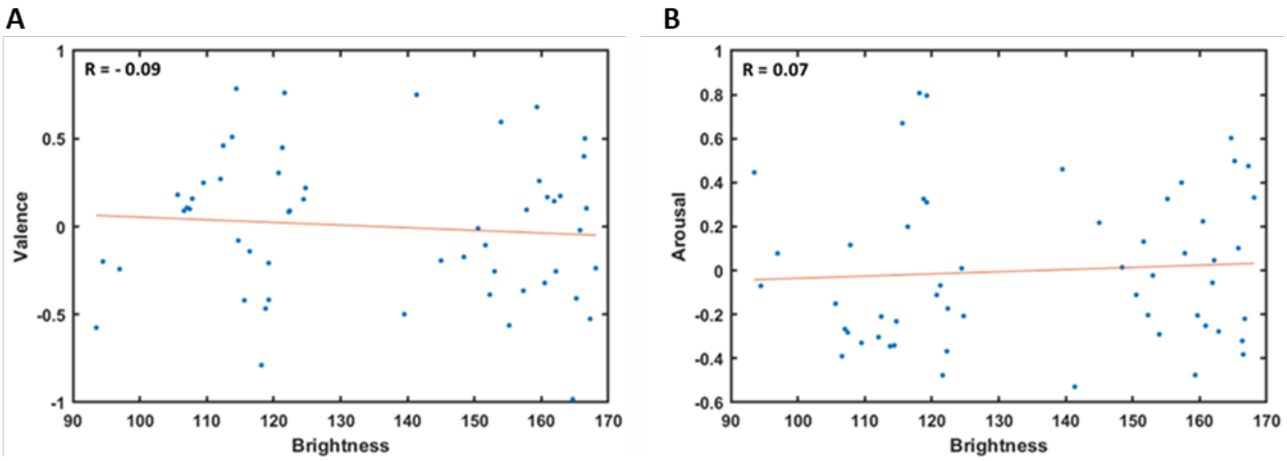

Figure S3. Architectures distribution within the space of valence and brightness (Panel A) and arousal and brightness (Panel B). The Pearson's linear correlation coefficient is reported on the top of both panels. Red lines indicate the best linear fit between the subjective ratings of valence (Panel A) and arousal (Panel B) and the brightness value of the architectures.

Considering that a parameter impacting on individual’s affective state could be the relative sill height to the eye level of the participant rather than to the location of the ceiling, we performed a correlation analysis between subjective valence and arousal ratings and the sill height relative to the subject’s eye level.

We computed the relative sill height as the average between the sill height of the second and third nucleus, scaled by the average participants’ eye level (1.60 m).

$$Relative\ Sill\ Height_i = \frac{Sill\ Height_{2°\ Nucelus,i} + \ Sill\ Height_{3°\ Nucelus,i}}{2} - 1.60$$

Where i identifies each of the specific architecture. The 54 combinations of architectural design resulted in 7 different relative sill heights (-1.075 m, -0.7 m, -0.325 m, -0.1 m, 0.5 m, 0.875 m, 2.075 m). Negative values indicated a sill height below the participant's eye level.

Pearson’s correlation coefficient was computed for the correlation analysis. The correlation between subjective ratings of valence and the relative sill height was found to be not significant ($p = 0.11$). Instead, subjective ratings of arousal and relative sill heights were found to be positively correlated ($p = 0.004$). However, the Pearson’s correlation coefficient revealed a moderate effect ($R = 0.385$). The figure below illustrates the results.

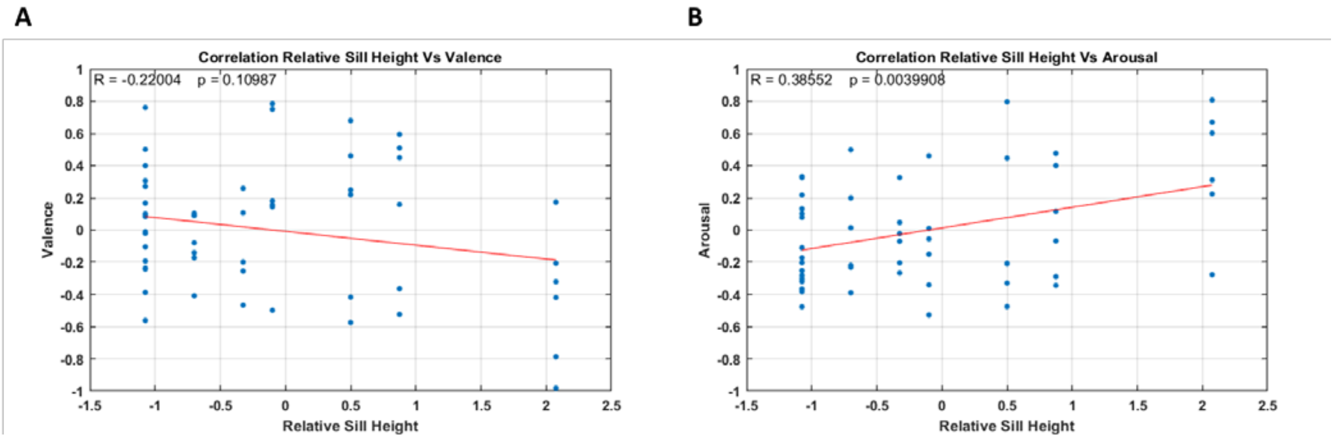

Figure S4. Architectures distribution considering valence (arousal) ratings and the relative sill height are illustrated in Panel A (Panel B). The Pearson's linear correlation coefficient and the p-value are reported on the top of both panels. Red lines indicate the best linear fit between the subjective ratings of valence (Panel A) and arousal (Panel B) and the relative sill height.

We conducted two separate Kruskal Wallis tests to further evaluate possible differences in the subject's preferences according to the relative sill heights. The null hypothesis was that subject's valence (and arousal) ratings belonged to the same distribution, not depending on the relative sill height. Results showed no significant differences among the 7 levels of relative sill heights, for both valence ($\chi2(6) = 9.651$, $p = 0.14$) and arousal ($\chi2(6) = 6.932$, $p = 0.327$) ratings.

Hence, although the present analysis returned a significant correlation between the relative sill height and the subjective arousal preference, we did not observe a clear preference for those architecture having windows vertically centred on, or slightly lower than, the eye-level of the participants, as hypothesized by the reviewer. In that case, we should have observed the highest (lowest) valence (arousal) subjective rating for the conditions where the relative sill height is close to zero (i.e., -0.325 m, 0.5 m). This condition is not verified.

However, with our virtual stimuli is not possible to effectively disentangle the combined effect due to the progressive variation of the sill height between consecutive nuclei and their relative position to the eye level of the participants. For instance, conditions relative to constant sill height across nuclei are missing.

Considering that valence and arousal ratings are not segregated in two sets but are distributed continuously between the two quadrants, it could be useful to make a more refined clustering. For this reason, we decided to perform an additional k-means cluster analysis, setting $k = 3$, thus identifying a new "Neutral" group of architectures. From the results it is possible to observe that the $k = 3$ cluster analysis confirmed the results obtained with $k = 2$, i.e., the sidewalls distance is still the only experimental condition according to which architectures were unbalanced between clusters ($\chi2 = 30.9$, $p = 3.2 *10-6$): the 78% of architectures with increasing sidewalls distance belongs to the LAPV cluster, while the 55% of the decreasing sidewalls distance architectures belong to the HANV cluster. Clustering architectures according to the factors Windows, Ceiling and Color did not return statistically significant results. The prevalence of architectures within the two clusters according to their experimental condition can be found in the Table below, along with the Figure S5 illustrating the results of the cluster analysis.

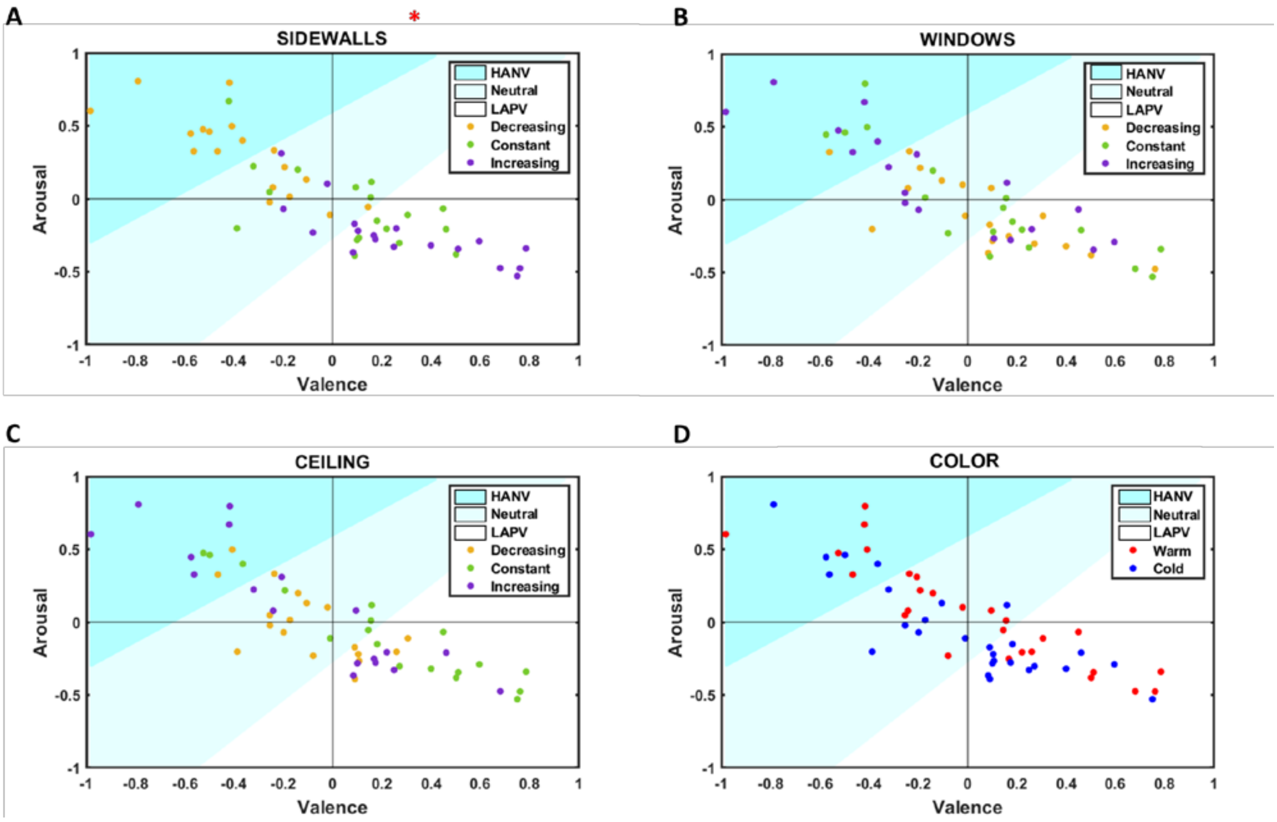

Figure S5. For each panel, the darker blue section of the plane includes architectures belonging to the HANV cluster, the lighter blue one comprises architectures within the Neutral cluster and the white one includes architectures belonging to the LAPV. Panel A, B, C: yellow, green, and purple dots identify architectures with decreasing, constant and increasing conditions for SideWalls, Windows, and Ceiling factors, respectively. Panel D: blue and red dots identify architectures with cold and warm texture color, respectively. The red asterisk indicates statistically significant results.

		HANV	Neutral	LAPV
SideWalls	Decreasing	55.56	44.44	0
	Constant	5.56	38.89	55.56
	Increasing	0	22.20	77.78

		HANV	Neutral	LAPV
Windows	Decreasing	5.56	44.44	50
	Constant	22.22	27.78	50
	Increasing	33.33	33.33	33.33

		HANV	Neutral	LAPV
Ceiling	Decreasing	11.11	55.56	33.33
	Constant	16.67	27.78	55.56
	Increasing	33.30	22.22	44.44

		HANV	Neutral	LAPV
Color	Warm	22.22	40.74	37.04
	Cold	18.52	29.63	51.85

Table S1. Percentage of architecture distribution within the clusters HANV, Neutral, and LAPV according to the experimental conditions.
